# P-1951. Incidence of SARS-CoV-2 Infection among those 6 Months to 49 Years of Age in the Cascadia Prospective Cohort Study, Oregon and Washington, United States, July 1, 2022 – March 31, 2024

**DOI:** 10.1093/ofid/ofae631.2110

**Published:** 2025-01-29

**Authors:** Mark A Schmidt, Neil D Yetz, Teresa Kimes, Cassandra Boisvert, Stephen P Fortmann, Holly C Groom, Jennifer L Kuntz, Richard A Mularski, Sacha L Reich, Ning Smith, Zack Acker, Sarah N Cox, Brenna Ehmen, Collrane J Frivold, Luis Gamboa, Sally Grindstaff, Peter D Han, Alex Harteloo, Tara Hatchie, Madison R Hollcroft, Natalie K Lo, Christina Lockwood, Kathryn McCaffrey, Lani Regelbrugge, Lea Starita, Jeremy Stone, Ana A Weil, Melissa Briggs-Hagen, Leora R Feldstein, Claire Midgley, Ian D Plumb, Sharon Saydah, Janet A Englund, Hanna Grioni, Helen Y Chu, Allison L Naleway

**Affiliations:** Center for Health Research, Kaiser Permanente Northwest, Portland, Oregon; Kaiser Permanente Center for Health Research, Lake Oswego, Oregon; Kaiser Permanente Northwest Center for Health Research, Portland, Oregon; Kaiser Permanente, Portland, Oregon; Kaiser Permanente, Portland, Oregon; Kaiser Permanente Center for Health Research, Lake Oswego, Oregon; Kaiser Permanente Center for Health Research, Lake Oswego, Oregon; 1. Kaiser Permanente Center for Health Research, Portland, Oregon, Portland, Oregon; Kaiser Permanente Center for Health Research, Lake Oswego, Oregon; Kaiser Permanente Center for Health Research, Lake Oswego, Oregon; Brotman Baty Institute, Seattle, Washington; University of Washington, Seattle, Washington; Brotman Baty Institute, Seattle, Washington; University of Washington, Seattle, Washington; Brotman Baty Institute for Precision Medicine, Seattle, Washington; Brotman Baty Institute, Seattle, Washington; University of Washington, Seattle, Washington; University of Washington, Seattle, Washington; University of Washington, Seattle, Washington; University of Washington School of Medicine, MOUNTLAKE TERRACE, Washington; University of Washington, Seattle, Washington; University of Washington, Seattle, Washington; Brotman Baty Institute, Seattle, Washington; Brotman Baty Institute, Seattle, Washington; University of Washington, Seattle, Washington; Brotman Baty Institute, University of Washington, Seattle, Washington; University of Washington, Seattle, Washington; Centers for Disease Control and Prevention, Atlanta, Georgia; Centers for Disease Control and Prevention, Atlanta, Georgia; Centers for Disease Control and Prevention, Atlanta, Georgia; Division of Foodborne, Waterborne, and Environmental Diseases, Centers for Disease Control and Prevention, Atlanta, GA, Atlanta, Georgia; Centers for Disease Control and Prevention, Atlanta, Georgia; Seattle Children’s Hospital, Seattle, Washington; Seattle Childrens Hospital, Seattle, Washington; University of Washington, Seattle, Washington; Kaiser Permanente Center for Health Research, Lake Oswego, Oregon

## Abstract

**Background:**

With the ending of the public health emergency and changes in the testing landscape, less is known about SARS-CoV-2 incidence in the community than earlier in the pandemic. Our goal was to describe SARS-CoV-2 incidence among individuals 6 months to 49 years of age enrolled in the Cascadia prospective community cohort study in Oregon and Washington, USA, by age, sex, and COVID-19 vaccination status.

**Figure:**
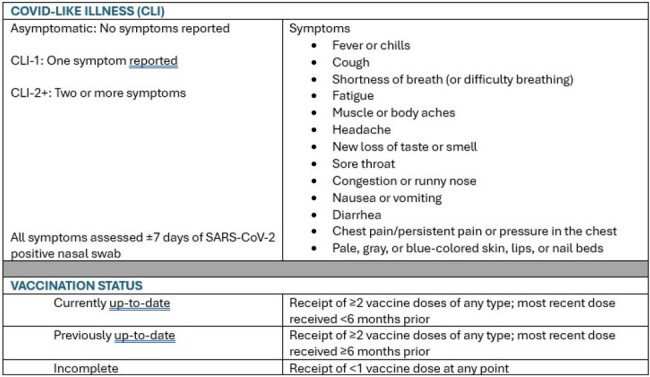

Covid-like illness (CLI) and vaccination status definitions

**Methods:**

We included Cascadia participants enrolled July 1, 2022 – March 31, 2024. We collected weekly nasal swabs and surveys from participants; more frequently among those with SARS-CoV-2 infection. We defined SARS-CoV-2 infection as a positive or inconclusive swab result and assessed covid-like illness (CLI) symptoms ±7 days from collection date (Fig.1). We followed individuals from receipt of their first nasal swab until the earliest of SARS-CoV-2 infection, loss to follow-up, or the end of the study period. We considered age and COVID-19 vaccination status (Fig. 1) as time-varying variables. We estimated incidence as the number of SARS-CoV-2 infections per 1,000 person-weeks (p-w) of observation, along with robust-error 95% confidence intervals (CI); we considered incidence rates to vary between strata if confidence intervals did not overlap.

Table

**Figure:**
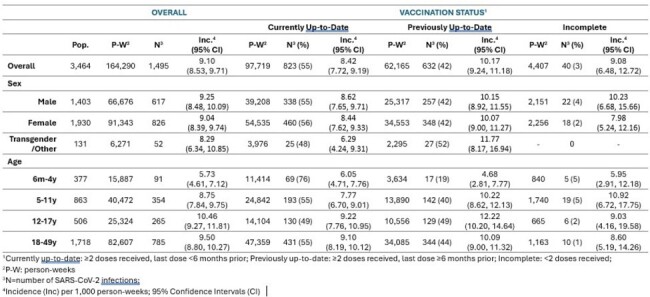

Incidence of SARS-CoV-2 Infection among Cascadia study participants, by sex, age, and vaccination status, July 1, 2022-March 31, 2024

**Results:**

We observed 1,495 SARS-CoV-2 infections among 3,464 included individuals with 164,290 p-w of follow-up; overall incidence rate was 9.10 (CI 9.09, 9.11). Incidence was similar by sex, lowest among those 6m-4y, and highest among those 12-17y (Table). Overall SARS-CoV-2 incidence was higher among those previously up-to-date (10.17 [CI 9.24, 11.18]) than among those currently up-to-date with vaccines (8.42 [CI 7.72, 9.19]; Table). A quarter (178/716) of SARS-CoV-2 infections among those < 18y were asymptomatic, compared with 10% (76/785) among those ≥18. The proportions reporting ≥2 CLI symptoms were 54% (49/91) among those 6m–4y, 62% (219/354) among those 5–11y, 69% (184/265) among those 12–17y, and 85% (666/785) among those 18–49y (Fig. 2).

**Figure:**
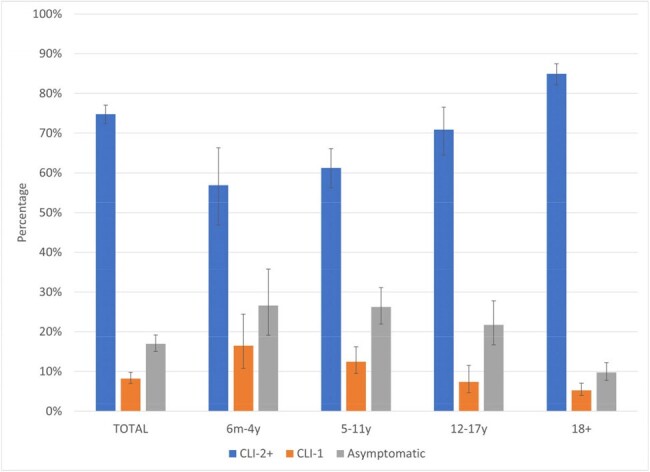

Frequency of covid-like illness (CLI) symptoms1 reported among Cascadia study participants, by age, July 1, 2022-March 31, 2024

**Conclusion:**

Our study fills a knowledge gap about the incidence of SARS-CoV-2 in the community later in the pandemic. Incidence was lowest among those recently vaccinated. These findings will inform continued discussions about COVID-19 vaccination strategies.

**Disclosures:**

Mark A. Schmidt, PhD, MPH, HilleVax: Grant/Research Support|Janssen: Grant/Research Support|Moderna: Grant/Research Support|Pfizer: Grant/Research Support|Vir Biotechnology: Grant/Research Support Neil D. Yetz, M.P.H., N/A: Expert Testimony Teresa Kimes, MS, Moderna: Grant/Research Support|Vir Biotechnology: Grant/Research Support Stephen P. Fortmann, MD, Pfizer: Grant/Research Support Holly C. Groom, MPH, Hillevax: Grant/Research Support|Moderna: Grant/Research Support Jennifer L. Kuntz, MS, PhD, Hillevax, Inc: Grant/Research Support Richard A. Mularski, MD, MSHS, MCR, Pfizer, Inc: Grant/Research Support Janet A. Englund, MD, Abbvie: Advisor/Consultant|AstraZeneca: Advisor/Consultant|AstraZeneca: Grant/Research Support|GlaxoSmithKline: Advisor/Consultant|GlaxoSmithKline: Grant/Research Support|Meissa Vaccines: Advisor/Consultant|Merck: Advisor/Consultant|Pfizer: Board Member|Pfizer: Grant/Research Support|Pfizer: Speaker at meeting|SanofiPasteur: Advisor/Consultant|Shinogi: Advisor/Consultant Helen Y. Chu, MD, MPH, Abbvie: Advisor/Consultant|Merck: Advisor/Consultant|Vir: Advisor/Consultant

